# Self-Difference Convolutional Neural Network for Facial Expression Recognition

**DOI:** 10.3390/s21062250

**Published:** 2021-03-23

**Authors:** Leyuan Liu, Rubin Jiang, Jiao Huo, Jingying Chen

**Affiliations:** 1 National Engineering Research Center for E-Learning, Central China Normal University, Wuhan 430079, China; lyliu@ccnu.edu.cn (L.L.); jrubin@mails.ccnu.edu.cn (R.J.); huojiao@mails.ccnu.edu.cn (J.H.); 2 National Engineering Laboratory for Educational Big Data, Central China Normal University, Wuhan 430079, China

**Keywords:** facial expression recognition, difference-based method, self-difference convolutional neural network, facial expression synthesis, facial expression classification

## Abstract

Facial expression recognition (FER) is a challenging problem due to the intra-class variation caused by subject identities. In this paper, a self-difference convolutional network (SD-CNN) is proposed to address the intra-class variation issue in FER. First, the SD-CNN uses a conditional generative adversarial network to generate the six typical facial expressions for the same subject in the testing image. Second, six compact and light-weighted difference-based CNNs, called DiffNets, are designed for classifying facial expressions. Each DiffNet extracts a pair of deep features from the testing image and one of the six synthesized expression images, and compares the difference between the deep feature pair. In this way, any potential facial expression in the testing image has an opportunity to be compared with the synthesized “Self”—an image of the same subject with the same facial expression as the testing image. As most of the self-difference features of the images with the same facial expression gather tightly in the feature space, the intra-class variation issue is significantly alleviated. The proposed SD-CNN is extensively evaluated on two widely-used facial expression datasets: CK+ and Oulu-CASIA. Experimental results demonstrate that the SD-CNN achieves state-of-the-art performance with accuracies of 99.7% on CK+ and 91.3% on Oulu-CASIA, respectively. Moreover, the model size of the online processing part of the SD-CNN is only 9.54 MB (1.59 MB ×6), which enables the SD-CNN to run on low-cost hardware.

## 1. Introduction

Facial expression recognition (FER) aims to classify a static face image or a sequence of face images into one of the typical facial expression classes, such as anger, disgust, fear, happiness, sadness, and surprise [1]. Facial expressions often convey cues about the emotional state and even the intentions of human beings. It is, therefore, unsurprising that automatic FER systems have many practical applications, including, but not limited to, human-robot interaction [2], leaner’s interest analysis [3,4], and detection of mental disorders [5]. Thereby, FER has become one of the hottest research topics in the computer vision community.

**Figure 1:**
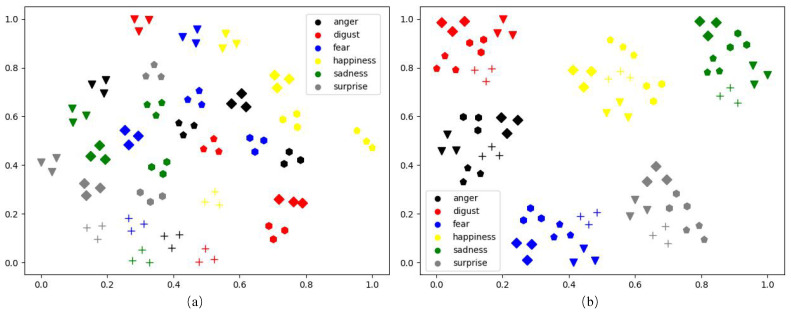
Visualization of deep features output by the fine-tuned VGG-face [8] (**a**) and the self-difference features extracted by the proposed self-difference convolutional network (SD-CNN) (**b**). Each dot represents the deep feature extracted from a face image. Different shapes denote different subject identities, and different colors represent different facial expressions.

Benefiting from the development of deep learning, great progresses have been made in the field of FER in the past decade [6]. However, FER is still a challenging problem due to the intra-class variation caused by subject identities. It is rather difficult to transfer face images into a feature space where the distance between two samples of different subjects with the same facial expression is always smaller than the distance between two samples of the same subject with different facial expressions. To better understand the intra-class variation, we picked out 90 samples of 5 randomly selected subjects (each subject with the 6 typical facial expressions and 3 expression intensities) from the CK+ dataset [7], and visualized the deep features of these samples extracted by the well-known VGG-face network [8] fine-tuned on CK+ using the t-SNE [9]. As shown in Figure 1a, the samples from all the six facial expression classes are entangled with each other, while most of the samples are relatively clustered according to their identities. As most of the samples distribute in the feature space according to the identities rather than facial expressions, it is difficult to design a high-precision classifier for FER.

To address the intra-class variation issue, several studies [10,11,12] have tried to recognize facial expression by comparing a subject’s expression with the neutral expression of the same subject. In our previous work [10], a deep feature extraction network called deep peak-neutral difference is proposed to use the difference between two deep representations of the fully expressive (peak) and neutral facial expression frames for FER. The peak-neutral-difference-based methods assume that the images with neutral and peak expressions can be discriminated accurately from the facial expression sequences. However, discriminating the neutral or peak expressions is also a tough task. Furthermore, facial expression sequences even may not obtainable in many application scenarios. In order to utilize the peak-neutral-difference approach under such scenarios, Yang et al. [12] treated facial expression as a combination of an expression component and a neutral component (or identity component) of a subject and proposed a De-expression Residue Learning (DeRL) procedure to eliminate the subject identity information. First, the DeRL uses a generative adversarial network to generate the corresponding neutral face image for the input image. Then, given the generated neutral face image, the DeRL learns the expression component deposited in the generative model for FER. Although the DeRL can generate a neutral expression image based on the single input image and tries to remove the identity component, the expression component deposited in the generative model is not powerful enough for facial expression classification.

In this paper, we propose a novel approach called Self-Difference Convolutional Neural Networks (SD-CNN) for FER. Our SD-CNN still uses the feature-level differences between the testing image and the corresponding synthesized expressions of the same subject for FER. However, unlike the existing difference-based FER methods that pick out or synthesize only one reference image with the neutral expression, our SD-CNN uses a conditional Generative Adversarial Network (cGAN) [13] to generate six reference images based on action unit intensities. Each of the generated reference images with one of the six typical facial expressions. Moreover, six compact and light-weighted difference-based CNNs, called DiffNets, are designed for classifying facial expressions. Each DiffNet extracts a pair of deep features from the testing image and one of the six synthesized reference images, and compares the difference between the deep feature pair. In this way, any potential facial expression in the testing image has an opportunity to be compared with the synthesized “Self”—an image of the same subject with the same facial expression as the testing image. Our insight is that the distance between any potential facial expression and the synthesized “Self” is very small. As the self-difference features extracted from the images with the same facial expression gather tightly in the feature space, the intra-class variation issue is alleviated and, thus, can improve the performance of FER. To confirm this insight, the self-difference features of the 90 samples mentioned above are also visualized using the t-SNE [9]. As illustrated in Figure 1b, the self-difference features of different subjects with the same facial expression are obviously clustered in the low-dimensional visualization space. It means that the self-difference features are discriminative for facial expression classification. Our SD-CNN has been evaluated on two widely-used FER datasets: CK+ [7] and Oulu-CASIA [14]. Experimental results show that our SD-CNN achieves high accuracies of 99.7% on CK+ and 91.3% on Oulu-CASIA, and it outperforms most of the current state-of-the-art FER methods on both the two datasets. Moreover, the model size of the online processing part of our SD-CNN is only 9.54 MB because of the compact and lightweight design of DiffNets.

The main contribution of this paper is three-fold:

(1) We propose a novel approach called SD-CNN for FER. The self-difference feature output by our SD-CNN can significantly alleviate the intra-class variation issue in FER an is discriminative for fical expression classification.
(2) We present a cGAN-base facial expression generator to synthesize reference images with the six typical facial expressions. Conditioned by empirical action unit intensities, the generator can synthesize photo-realistic images with the desired expressions from any input face image.
(3) We design networks called DiffNets to extract the self-difference feature between the testing image and the synthesized expression image. Despite the fact that DiffNets are compact and light-weighted, they achieve state-of-the-art performance on public FER datasets.

The rest of the paper is arranged as follows: the related work is introduced in Section 2, the details of the SD-CNN are described in Section 3, the experiments are presented in Section 4, and the conclusions are given in Section 5.

## 2. Related Work

FER methods can be divided into two main categories according to the information used: static image-based FER and sequence-based FER. Static image-based methods extract visual features only from a single face image, while sequence-based methods learn representations from a sequence of face images with contiguously changed expressions and may utilize the spatial-temporal information among frames.

### 2.1. Static Image-Based FER Methods

To mitigate the overfitting problem when training FER models with relatively small-scale facial expression data, many studies fine-tune networks that have been pre-trained on task-related large-scale datasets. Liu et al. [15] proposed to pre-train the VGG-face [8] on a large-scale face-recognition dataset and then fine-tuned the network on a small facial expression dataset for FER. To extend the generalizability of FER models, Mollahosseini et al. [16] fine-tuned a GoogleLeNet-like [17] network on a set of public available facial expression datasets. Ding et al. [18] presented a method called FaceNet2ExpNet to transform visual representations from the face recognition domain into the facial expression domain. The convolutional layers of the FaceNet2ExpNet are first trained and regularized by an off-the-shelf face recognition network, then the whole FaceNet2ExpNet, which consists of the pre-trained convolutional layers and fully-connected layers, is re-trained jointly on facial expression datasets. The FaceNet2ExpNet achieves an accuracy of 98.6% on the small-scale CK+ dataset when following a 10-fold cross-validation protocol. Although pre-training FER networks on other face-related datasets help improve the performance, the identity information retained in the pre-trained models may have a negative impact on the accuracy of FER [10].

To address the identity retaining problem, several researchers proposed to subtract the identity component from the face image. In order to develop a pose-invariant facial expression recognition algorithm, Zhang et al. [19] presented a GAN-based model to synthesize face images with different poses and expressions. In Reference [20], a GAN-based approach, called Identity-Adaptive Generative model (IA-gen), is proposed to alleviate the identity retaining issue by minimizing the distance between the synthesized prototypic facial expressions and the query image. Based on a CNN fine-tuned on FER datasets, the IA-gen achieves accuracies of 96.75% on CK+ and 88.92% on Oulu-CASIA. Fabiano et al. [21] first utilized a deep-learning-based 3D morphable model to synthesize 3D images with different expressions, and then employed the synthesize 3D images to train deep neural networks for facial emotion recognition on 3D datasets.

Recently, many researchers embedded the attention mechanism into FER networks to make the networks focus on the most expressive facial regions. In order to suppress the uncertainties caused by low-quality face images and ambiguous facial expressions, Wang et al. [22] proposed a Self-Cure Network (SCN) to weight each sample in training dataset with a ranking regularization by embedding a self-attention mechanism into neural networks. The SCN outperforms most of the state-of-the-art methods on several public FER datasets. Wang et al. [23] proposed a Region Attention Network (RAN), which employs a region biased loss to encourage high attention weights for the important face regions, to make the network extracts the most expression-related features for FER. Recently, Gan et al. [24] proposed to simulate the coarse-to-fine attention mechanism of human beings, and developed a multiple attention network to boost the performance of facial expression recognition. However, this multiple attention network only achieves an accuracy of 96.4% on the CK+ dataset.

In order to apply automatic FER systems on smart-phone and other embedded platforms, many efforts have been made to design compact and light-weighted neural networks for FER. In order to train a FER classifier with a lower requirement of computing resources, Zhu et al. [25] designed a neural network called IExpressNet-based on the ResNet-18. The IexpressNet is trained in an incremental manner and, thus, reduces the time consumption by over 80%. Zeng et al. [26] developed a FER network that consists of only 3 convolutional layers. Although this network is rather compact, the best accuracy on the CK+ dataset achieved by it is 95.79%, which is much lower than the deeper FER networks, like FaceNet2ExpNet [18] and DeRL [12].

### 2.2. Sequence-Based FER Methods

In order to reduce the difficulty of facial expression recognition, most sequence-based methods focus on utilizing frames with the most expressive (peak) expressions for FER. Zhao et al. [11] proposed a peak-piloted deep network (PPDN) that embeds the facial expression evolution from non-peak to peak frames into the network parameters. By using a special-purpose back-propagation procedure called PGS for network optimization, the PPDN drives the intermediate-layer features of the non-peak expression sample towards those of the peak expression sample. Yu et al. [27] proposed a deeper cascade peak-piloted network (CPPN) to employ the most expressive images for boosting the performance of the weak facial expression recognition. Similar to the PPDN [11], the CPPN also uses the peak expression to supervise the non-peak expression of the same subject. Despite the fact that CCPN achieves an accuracy of 99.30% on the CK+ dataset, the network of CCPN is 42 layers deep.

Many studies exploited both the spatial and temporal information to to improve the performance of facial expression recognition. To extract representative features from facial expression sequence, Chen et al. [28] proposed a framework named Facial Motion Prior Networks (FMPN) to employ domain knowledge for FER. The insight of the FMPN is that focusing on facial muscle moving regions can help to extract representative features and, thus, improve the FER performance. In order to improve the performance of FER by exploiting more information, Jung et al. [29] proposed to train two CNN-based networks joinlty to squeeze spatial-temporal information form the facial expression sequences and the temporal facial landmark points, respectively. Kuo et al. [30] proposed a compact CNN-based (CompactCNN) approach that employs the gated recurrent units to exploit temporal information among frames. By exploiting the temporal information, the performance of the CompactCNN is boosted from 97.37% to 98.47% on the CK+ dataset. Recently, Meng et al. [31] developed an end-to-end framework called Frame Attention Networks (FAN) to aggregate the spatial features among frames with temporal information. The FAN first employs a spatial attention module to learn an attention weight for each frame, then aggregate the features extracted from all the frames to form a single representation adaptively based on the attention weight of each frame. As a result, the FAN achieves the state-of-the-art performance on the CK+ dataset with an accuracy of 99.68%. Despite the fact that performance of FER can be improved by squeezing spatial-temporal information among frames, facial expression sequences are not always obtainable in many application scenarios.

## 3. Methodology

### 3.1. Overview

**Figure 2:**
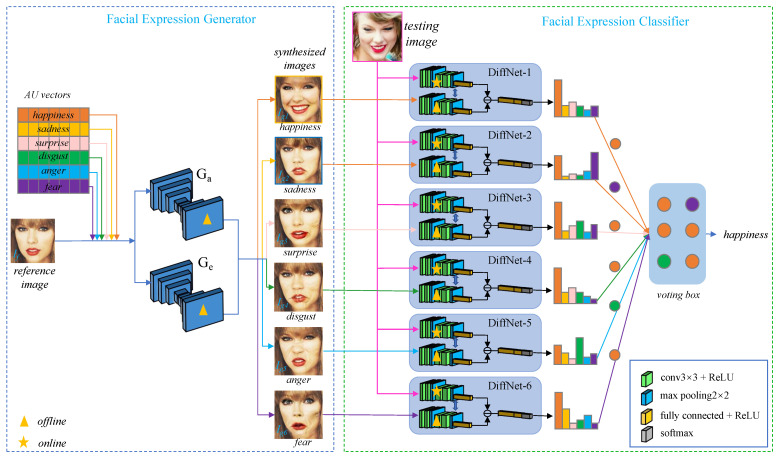
Framework of the proposed facial expression recognition method. The proposed method which at its core consists of two modules, i.e., the facial expression generator and the facial expression classifier. The facial expression generator synthesizes photo-realistic images with the six typical facial expressions from an input face image under an arbitrary facial expression. The facial expression classifier uses six DiffNets for facial expression classification.

The proposed FER method consists of two main modules, i.e., the facial expression generator and the facial expression classifier, as illustrated in Figure 2. Given the empirical Action Unit (AU) vectors of facial expressions, the facial expression generator employs a cGAN [13,32] to synthesize photo-realistic reference images with the six typical facial expressions (i.e., happiness, sadness, surprise, disgust, anger, and fear) from an input face image under an arbitrary facial expression. The facial expression classifier is composed of six DiffNets. Each DiffNet first extracts a pair of deep features from the testing image and one of the synthesized reference images, respectively, by two subnets named the query-subnet and the reference-subnet; then produces a difference vector by comparing the features extracted by these two subnets; and finally outputs a probability distribution over predicted facial expressions. As the six DiffNets output probability distributions over predicted facial expressions for each testing image, a voting scheme is adopted to make the final decision. In our voting scheme, each DiffNet holds a vote and uses a “winner-take-all” scheme to cast its vote. After being voted by all of the six DiffNets, the testing image is classified as the facial expression class that has received the most votes.

The networks in our method are divided into the offline components and the online components according to run-time. As illustrated in Figure 2, only the query-subnets in the DiffNets need to process online, while all the other networks can run offline. Since each query-subnet consists of only 4 light-weighted convolutional layers and 3 narrow fully-connected layers, our method is able to run on devices with low-cost processors and memory.

### 3.2. Facial Expression Generator

**Facial Expression Representation.** The aim of our facial expression generator is to synthesize photo-realistic images with desired facial expressions from an input face image under an arbitrary facial expression. Recently, Pumarola et al. [32] proposed a deep network architecture for synthesizing facial expressions by a novel GAN conditioning scheme based on facial action units. Inspired by this work, we also employ a cGAN conditioned by facial action units to synthesize the six typical facial expressions, including anger, disgust, fear, happiness, sadness, and surprise. For this purpose, each facial expression is encoded by a vector which consists of a set of *K* action unit intensities:

(1) $$V_{i}={\left(v_{i1},\cdots ,v_{ik},\cdots ,v_{iK}\right)}^{{}^{T}},$$

where i∈{1,⋯,6} is the index for each of the six typical facial expressions, and $v_{ik}\in \left[0,1\right]$ denotes the normalized intensity of the *k*th action unit. According to the Facial Action Coding System (FACS) [33], each of the six facial expressions can be represented by a vector of empirical action unit intensities that: ${\tilde{V}}_{i}={\left({\tilde{v}}_{i1},\cdots ,{\tilde{v}}_{iK}\right)}^{T}$. In our implementation, empirical action unit intensities are calculated from the EmotioNet dataset [34]. To make the generated facial expressions more diverse, we sample an action unit vector for each subject from the empirical action unit intensities using the Gaussian distribution.

(2) $$v_{ik}∼N\left({\tilde{v}}_{ik},\sigma_{ik}^{2}\right),$$

where N denotes the Gaussian distribution, and $\sigma_{ik}^{2}$ is a given variance.

**Figure 3:**
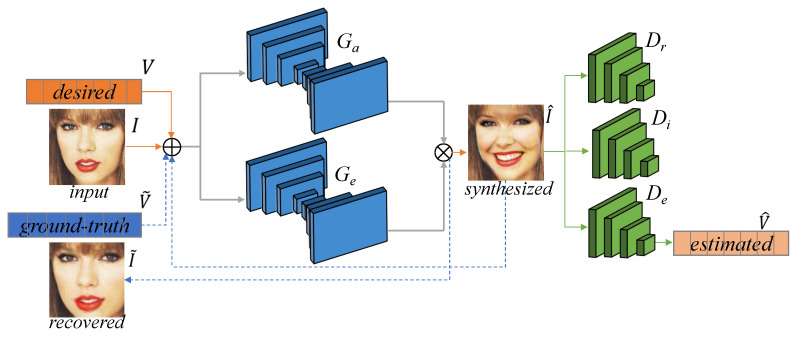
Network structure of the facial expression generator.

**Network Structure.** As illustrated in Figure 3, the network structure of our cGAN-based facial expression generator mainly consists of the generative networks *G* and the discriminative networks *D*. The generative networks are designed to synthesize a photo-realistic image $\hat{I}$ with the desired facial expression $V_{i}$ from the input image *I*. In order to make the generative networks focus exclusively on the image regions that are responsible of synthesizing the desired facial expression, the attention mechanism is embedded into the generative networks [32]. Concretely, the generative networks are composed of an attention network $G_{a}$ and an expression synthesis network $G_{e}$, that is, $G=(G_{a},G_{e})$. The output of $G_{a}$ is a pixel-wise mask that indicates the contribution of each pixel to the desired facial expression. Then, the synthesized image is produced by the outputs of the two networks and the original input image: $\hat{I}=G_{a}\left(I|V_{i}\right)G_{e}\left(I|V_{i}\right)+\left[1-G_{a}\left(I|V_{i}\right)\right]I$. In order to utilize the unsupervised strategy to train the facial expression generator, the generative networks are applied twice. First, we apply the generative networks to transform the input image *I* to the synthesized image $\hat{I}$ with the desired facial expression $V_{i}$: $\hat{I}=G\left(I|V_{i}\right)$; then, we adopt them again to recover the input image from the synthesized image $\hat{I}$ using the ground-truth facial action unit intensities ${\tilde{V}}_{i}$: $\tilde{I}=G\left(\hat{I}|{\tilde{V}}_{i}\right)$, where $\tilde{I}$ denotes the recovered image. By using this strategy, the generator does not require supervision, i.e., no pairs of images of the same person with different expressions, nor the target image $\hat{I}$ are assumed to be known. The discriminative networks *D* are composed of a reality judgement network $D_{r}$, which valuates the synthesized image in its photo-realism, an identity verification network $D_{i}$ which verifies the input image and the synthesized image are the same individual, and an expression estimation network $D_{e}$ which estimates the action unit intensities ($\hat{V}$) of the synthesized image.

**Loss Functions** Our facial expression generator is trained following a min-max game:

(3) $$G^{*}=\arg\min_{G}\max_{D}L_{G},$$

where $L_{G}$ is the loss function which consists of five parts: the adversarial loss ($L_{d}$), the attention loss ($L_{a}$), the identity loss ($L_{i}$), the perceptual loss ($L_{p}$), and the expression loss ($L_{e}$). The adversarial loss proposed by WGAN-GP [35] is employed:

(4) $$\begin{array}{cc} L_{d}= & (E_{I}\left[D_{r}\left(G\left(I|V_{i}\right)\right)\right]-E_{I}\left[D_{r}\left(I\right)\right])+\\ & \lambda_{1}E_{\dot{I}}\left[{\left(\left|\right|\nabla_{\dot{I}}D_{r}\left(\dot{I}\right){\left|\right|}_{2}-1\right)}^{2}\right], \end{array}$$

where $\dot{I}$ is the input image with random noise, and $\lambda_{1}$ is a penalty coefficient. Following Reference [32], the attention loss is defined as:

(5) $$\begin{array}{cc} L_{a}= & E_{I}\left[\sum_{i,j}\left[{\left(\nabla_{x}G_{a}{\left(I|V_{i}\right)}_{i,j}\right)}^{2}+{\left(\nabla_{y}G_{a}{\left(I|V_{i}\right)}_{i,j}\right)}^{2}\right]\right]+\\ & \lambda_{2}E_{I}\left[|\right|G_{a}\left(I|V_{i}\right){\left|\right|}_{2}], \end{array}$$

where ∇ denotes the gradient of an image, and $\lambda_{2}$ is a penalty coefficient. The identity loss ($L_{i}$) is defined for identity verification:

(6) $$L_{i}=y_{1}log\left(q_{1}\right)+\left(1-y_{1}\right)log\left(1-q_{1}\right),$$

where $y_{1}=1$ if the input image and the synthesized image are predicted as the same individual by the identity verification network $D_{i}$, and $q_{1}$ denotes the probability that the input image and the synthesized image the same individual. The perceptual loss [36] is defined by the difference between the input image and the recovered image:

(7) $$L_{p}=E_{I}[||I-G\left(G\left(I|V_{i}\right)|{\tilde{V}}_{i}\right){\left|\right|}_{2}].$$

The expression loss is defined as:

(8) $$L_{e}=E_{I}\left[|\right|D_{e}\left(G\left(I|V_{i}\right)\right)-V_{i}{\left|\right|}_{2}]+E_{I}\left[|\right|D_{e}\left(I\right)-{\tilde{V}}_{i}{\left|\right|}_{2}].$$

Before training the whole facial expression generator, we pre-train the expression estimation network $D_{e}$ using the following simple loss function $L_{e2}$:

(9) $$L_{e2}=E_{I}\left[|\right|D_{e}\left(I\right)-{\tilde{V}}_{i}{\left|\right|}_{2}].$$

Finally, the whole loss function is built by combining all the four loss functions:

(10) $$L_{G}=\lambda_{d}L_{d}+\lambda_{a}L_{a}+\lambda_{i}L_{i}+\lambda_{p}L_{p}+\lambda_{e}L_{e},$$

where $\lambda_{d}$, $\lambda_{a}$, $\lambda_{p}$, and $\lambda_{e}$ are the hyper-parameters.

### 3.3. Facial Expression Classifier

**Figure 4:**
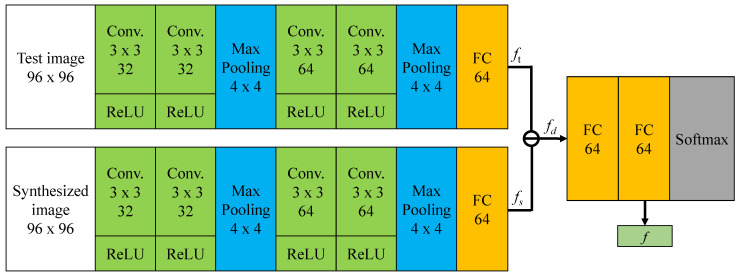
Network structure of the DiffNet for facial expression classification.

**The DiffNet.** The architecture of a DiffNet is given in full detail in Figure 4. A DiffNet consists of two branched subnets, i.e., the query-subnet which is used to extract features from the testing image, and the reference-subnet which is used to extract features from a synthesized reference image. The query-subnet and the reference-subnet are designed as two peer networks with the same structure, but they do not share parameters for each other. A query-subnet/reference-subnet comprises two convolutional units and a fully-connected layer. Both the two convolutional units are composed of two convolutional layers followed by a ReLU and a max pooling, but the numbers of their convolutional kernels are, respectively, 32 and 64. After processed by a subtraction operation, a feature-level difference vector between the testing image and the synthesized expression of the same subject is obtained. Based on the difference vector, the DiffNet uses two fully-connected layers and a Softmax for facial expression classification. Since the DiffNet is designed using a compact and light-weighted structure, the number of parameters, the model size, and the number of operations for a DiffNet are, respectively, 0.395 M, 1.59 MB, and 0.033G FLOPs.

**Self-Difference Feature.** As mentioned before, the facial expression classifier comprises six DiffNets, each of which compares the difference deep features extracted from the input image and one of the six synthesized reference expression images. Therefore, any potential facial expression in the input image has an opportunity to be compared with the synthesized “Self” facial expression. The distance between any potential facial expression and the synthesized “Self” is usually very small. Thus, the self-difference features extracted from the images with the same facial expression will gather in the feature space, which will alleviate the intra-class variation issue. Although compact and light-weighted DiffNets are employed for facial expression classification, the self-difference feature is discriminative enough.

**Loss Functions.** The loss function of each DiffNet is composed of two partial terms: the cross-entropy loss $L_{c}$ and the triplet loss $L_{t}$. The cross-entropy loss is defined as:

(11) $$L_{c}=-\frac{1}{M}\sum_{i=1}^{M}\sum_{k=1}^{6}y_{i}^{k}\log\left(p_{i}^{k}\right),$$

where *M* is the number of samples in a mini-batch, $y_{i}^{k}\in \left\{0,1\right\}$ is the ground-truth label that indicates whether the *i*th sample belongs to the *k*th facial expression class, and $p_{i}^{k}$ denotes the predicted Softmax probability that the *i*th sample belongs to the *k*th facial expression. The triplet loss [37], which is popularly used in the filed of person re-id, is also utilized to optimize our DiffNets. When training a DiffNet with the triplet loss, each randomly selected sample is called as the “anchor”. Except for the anchor, we randomly select two additional samples: one belongs to the same facial expression class as the anchor, while the other one with a different facial expression against the anchor. These two samples are, respectively, called as the “positive” and the “negative”. The deep features of these three samples output by the last fully-connected layer of a DiffNet are, respectively, denoted as $f^{a}$, $f^{p}$, and $f^{n}$. Thereby, a triplet is formed by $<f^{a},f^{p},f^{n}>$, and the triplet loss is defined using this triplet:

(12) $$\begin{array}{c} L_{t}=-\sum_{i=1}^{P}\sum_{a=1}^{K}[\max_{p=1\ldots K}\left|\right|f_{i}^{a}-f_{i}^{p}{\left|\right|}_{2}\\ -\min_{\begin{array}{c} j=1\ldots P\\ n=1\ldots K\\ j\neq a \end{array}}\left|\right|f_{i}^{a}-f_{j}^{n}{\left|\right|}_{2}{+m]}_{+} \end{array},$$

where *m* is the margin between the intra-class and inter-class, and ${\left[\cdot \right]}_{+}=max\left(\cdot ,0\right)$. In each mini-batch, we select *P* facial expression class and *K* images from each class. Since the positive pair $\left(f^{a},f^{p}\right)$ are extracted from the samples with the same facial expression, while the negative pair $\left(f^{a},f^{n}\right)$ are extracted from images with different facial expressions, the purpose of triplet loss is to make the intra-class distance as small as possible while making the inter-class distance as large as possible. Finally, the full loss function is defined by linearly combining the two loss terms:

(13) $$L_{C}=\lambda_{c}L_{c}+\lambda_{t}L_{t},$$

where $\lambda_{c}$ and $\lambda_{t}$ are the hyper-parameters that control the relative importance of the two loss terms.

**Voting Scheme.** As the six DiffNets in our facial expression classifier output six groups of probabilities for each testing image, a voting scheme is designed to make the final decision. In our voting scheme, each DiffNet holds a vote and uses a “winner-take-all” scheme to cast its vote. Formally, let ${\left\{p_{j}^{1},\cdots ,p_{j}^{k},\cdots ,p_{j}^{6}\right\}}_{j=1,\cdots ,6}$ denote the probabilities output by the Softmax layer of the *j*th DiffNet. Then, the *j*th DiffNet casts its vote to the $k^{*}$-th facial expression class:

(14) $$k^{*}=\underset{k}{\arg\max}\left\{p_{j}^{1},\cdots ,p_{j}^{k},\cdots ,p_{j}^{6}\right\}.$$

After being voted by all of the six DiffNets, the testing image is classified as the facial expression class that has received the most votes. If more than one class has received the same votes, then the final decision is made by the maximum Softmax probability output by the DiffNets that cause the tie.

## 4. Experimental Results

### 4.1. Implementation Details

The facial expression generator and the DiffNets in the facial expression classifier are trained independently. The expression generator is trained on 500,000 images randomly selected from the EmotioNet dataset [38]. The hyper-parameters for the partial loss functions in the generator are set as $\lambda_{d}=1$, $\lambda_{a}=0.1$, $\lambda_{p}=5$, $\lambda_{i}=5$, and $\lambda_{e}=4000$.

The six DiffNets are trained on the CK+ [7] and Oulu-CASIA [14] datasets following a 10-fold cross-validation protocol. We do not utilize the “pre-training then fine-tuning” scheme for training our models. In other words, the DiffNets have not been pre-trained on any other dataset. Since both the CK+ and Oulu-CASIA datasets consist of sequences/videos with continuous facial expressions range from neutral to peak, only the images whose sequence number is greater than 8 are captured for training. Each face in the images is cropped and resized to 128×128 pixels. In order to extend the training samples, half of the images in the original training dataset are randomly selected and augmented using FiveCrop [39] and horizontal flipping. The weight parameters of the partial loss functions for the DiffNets are set as $\lambda_{c}=1$, and $\lambda_{t}=10$. When training the DiffNets, the Adam with a momentum of 0.9 is adopted as the optimizer, and the mini-batch size is fixed to 64. Since each of the fully-connected layers only consists of 64 neurons, dropout is utilized to avoid overfitting. The dropout rate is set as 0.5. Each DiffNet is first trained independently for 1000 epochs, and then all the six DiffNets are jointly trained for another 500 epochs. All the deep neural networks in our method are implemented by PyTorch. It takes about 24 h for training all the six DiffNets on a computer with a single NVIDIA GeForce 2080 Ti GPU.

### 4.2. Datasets and Performance Metric

**Figure 5:**
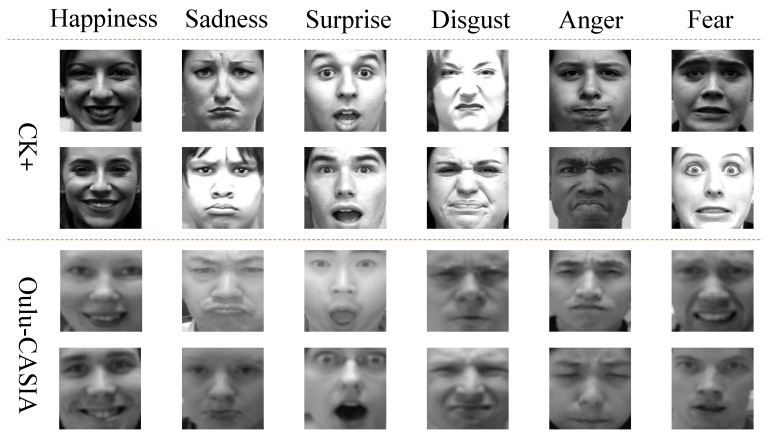
Example samples randomly selected from the CK+ [7] and Oulu-CASIA [14] datasets.

**Datasets.** The proposed SD-CNN is extensively evaluated on two widely-used FER datasets: CK+ [7] and Oulu-CASIA [14]. The CK+ dataset includes 593 sequences from 123 subjects, and involves seven facial expression classes (i.e., anger, contempt, disgust, fear, happiness, sadness, and surprise). In our experiments, the 18 sequences with contempt expression are ignored. The Oulu-CASIA dataset contains 2280 videos with the six typical facial expressions (i.e., anger, disgust, fear, happiness, sadness, and surprise) from 80 subjects. The videos in Oulu-CASIA are captured with two imaging systems, NIR (Near Infrared) and VIS (Visible light), but we only use the VIS videos in our experiments. As the sequences/videos in both CK+ and Oulu-CASIA record faces with facial expressions range form neutral to peak, we cut all the sequences/videos into images and ignore the images with neutral expressions. Totally, we capture 3482 images from the sequences in CK+ and 6540 images from the videos in Oulu-CASIA. As mentioned above, our experiments conduct on CK+ and Oulu-CASIA follow a 10-fold cross-validation protocol. The frames from one sequence are kept only either in the training set or in the testing set. When conducting experiments on CK+, 3134 images are used for training and 348 images are used for testing; when conducting experiments on Oulu-CASIA, 5886 images are used as the training dataset, and 654 images are used as the testing dataset. The class distribution of images in the CK+ and Oulu-CASIA datasets is listed in the Table 1. Some samples randomly selected from these two datasets are shown in Figure 5.

**Performance Metric.** The Accuracy (ACC) is adopted as the metric for evaluating the facial expression recognition performance:

(15) $$ACC=[\frac{1}{N}\sum_{i=1}^{N}\left({\hat{y}}_{i}==y_{i}\right)]\times 100\%,$$

where *N* is the number of samples in the testing dataset, and *y* and $\hat{y}$ are, respectively, the ground-truth label and the predicted facial expression class of a testing sample.

### 4.3. Experiments on CK+

Our SD-CNN method is compared with the recent state-of-the-art FER methods, including DeRL [12], FN2EN [18], FMPN [28], VGG-face [8], MicroExpNet [34], GoogLeNet [17], MultiAttention [24], DSAE [26], GCNet [40], DynamicMTL [41], IA-gen [20], CompactCNN [30], DTAGN(Joint) [29], CPPN [27], DPND [10], PPDN [11], and FAN [31]. Table 2 reports our experimental results and shows the comparisons with these methods. In Table 2, the methods are divided into two groups according to the information used: static image-based FER and sequence-based FER. It is can be seen that our method achieves the best performance among the static image-based methods with an accuracy of 99.7%, which surpasses the current state-of-the-art image-based method (i.e., the DeRL [12]) by 0.4%. Even though much less information is used, our method achieves very competitive performance with respect to the state-of-the-art sequence-based method (i.e., the FAN [31]). For a subject, our method only need a single image for facial expression recognition, but the FAN [31] needs to capture a sequence of images with facial expressions range from neural to peek. However, image sequence with different expression intensities for an individual subject is not always available in practice. Moreover, the high performance of sequence-based methods depends on the accuracy of expressive (peak) frames identification. As reported in Reference [27], when conducting experiments only on the images with weak expressions, the performance of sequence-based methods usually decreases sharply. For example, the accuracies of the PPDN [11] and the CPPN [27] on the weak expression images in CK+, respectively, decrease from 99.3% and 98.3% to 83.4% and 92.5%.

**Figure 6:**
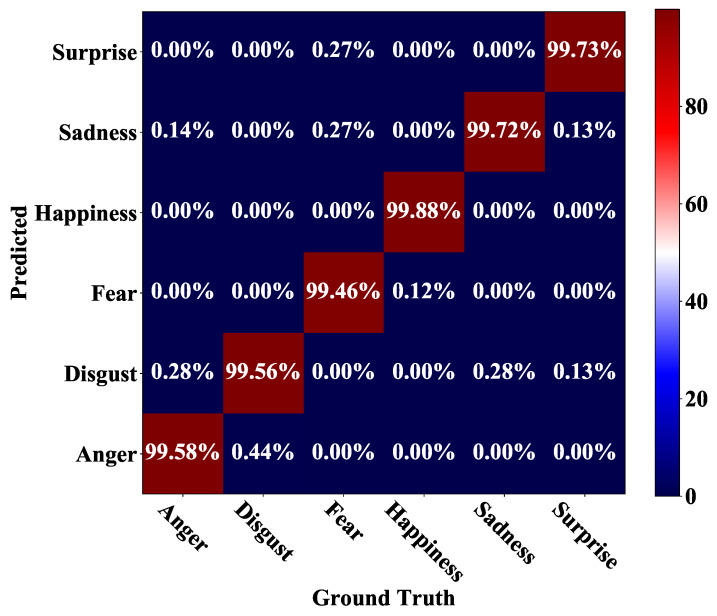
Confusion matrix for the CK+ dataset.

**Figure 7:**
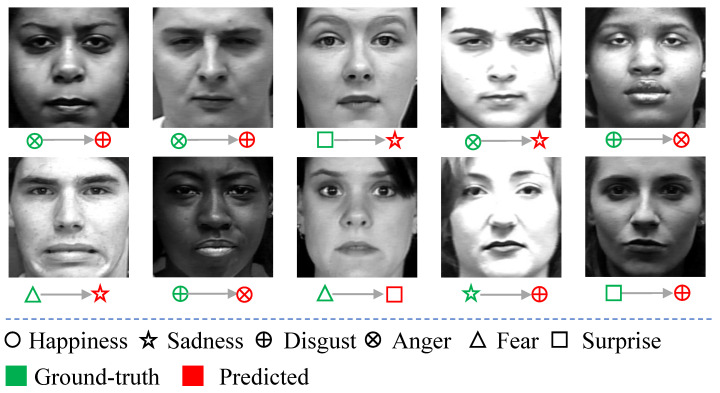
Testing samples in CK+ that are misclassified by our method.

Figure 6 shows the confusion matrix of our SD-CNN on the CK+ dataset. It can be observed that happiness, sadness, fear and surprise are almost recognized perfectly, with accuracies of 99.88%, 99.72%, 99.46%, and 99.73%, respectively. Anger and disgust are relatively hard to recognize. For the testing samples with anger, 0.28% of them are misclassified as disgust, and 0.14% of them are misclassified as surprise. For the testing samples with disgust expression, 0.44% of them are misclassified as anger. Figure 7 illustrates some testing samples in CK+ that are misclassified by our method. Obviously, most of these misclassified samples are with very weak expressions, which are rather hard to be classified correctly by human beings.

In order to evaluate the effect of the facial expression generator’, we use only 1 DiffNet to compare the test image with the reference (neutral expression) image and the performance on CK+ decreases from 99.7% to 92.4%.

**Figure 8:**
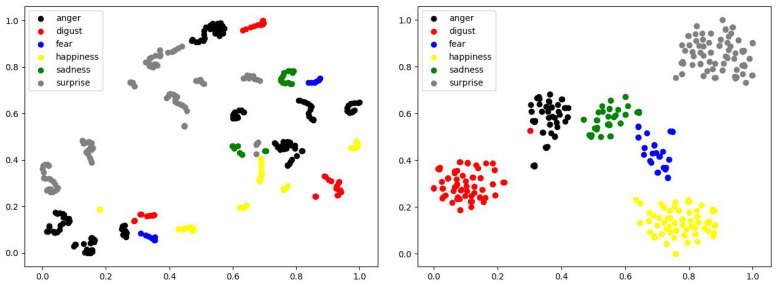
Visualization of deep features output by the VGG-face [8] (**left**) and our method (**right**) on the CK+ dataset. Each dot represents the deep feature extracted from a testing sample.

To better understand why our method achieves good performance, we visualize the self-difference features extracted by our SD-CNN and deep features extracted by the fine-tuned VGG-face [8] using t-SNE [9]. As shown in Figure 8, the self-difference features output by our method are closely gathered according to their facial expression classes in the two-dimensional visualization space, while the deep features output by the VGG-face are entangled with each other, and there is obvious confusion among different classes.

### 4.4. Experiments on Oulu-CASIA

Our SD-CNN is also compared with the recent state-of-the-art methods, including FN2EN [18], DeRL [12], GoogLeNet (fine-tuned) [17], VGG-face (fine-tuned) [8], GCNet [40], DynamicMTL [41], MicroExpNet [34], MultiAttention [24], IA-gen [20], PPDN [11], DPND [10], DTAGN(Joint) [29], and CompactCNN [30], on the Oulu-CASIA dataset. As shown in Table 3, our method achieves the highest accuracy of 91.3 %, which outperforms the state-of-the-art static image-based method (i.e., the DynamicMTL [41]) by 1.7% and also suppresses the state-of-the-art sequence-based method (i.e., the CompactCNN [30]) by 2.7%.

**Figure 9:**
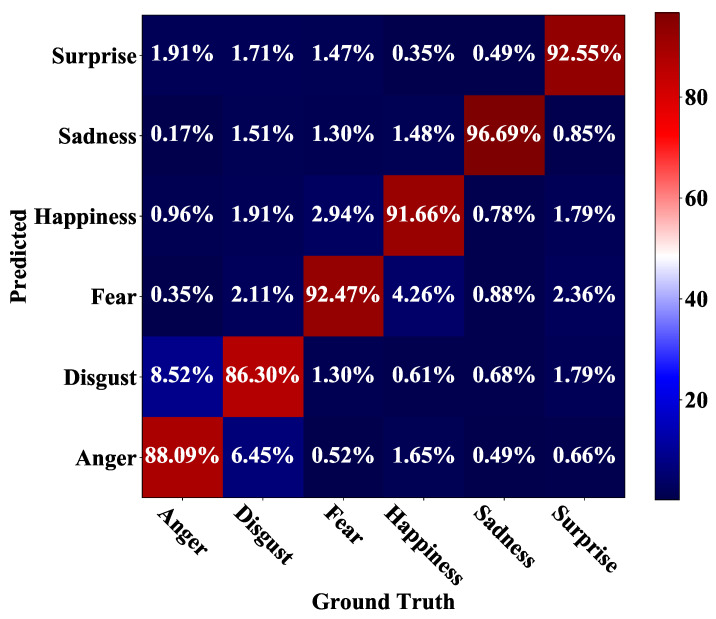
Confusion matrix for the Oulu-CASIA dataset.

**Figure 10:**
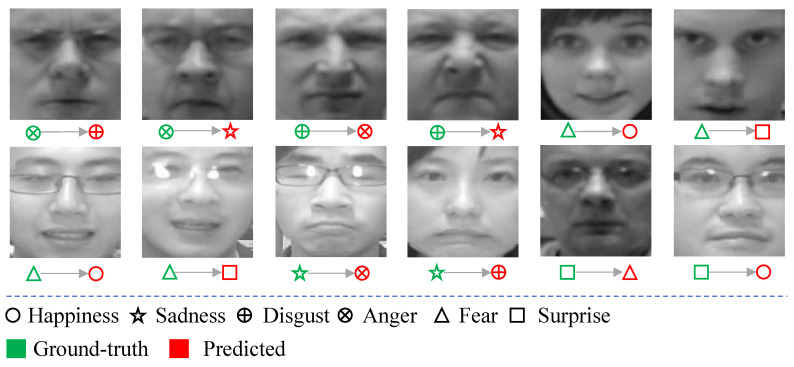
Testing samples in Oulu-CASIA that are misclassified by our method.

Figure 9 shows the confusion matrix for the Oulu-CASIA dataset. Among the six facial expressions in this dataset, sadness, happiness, surprise, and fear are recognized with high accuracies of over 90%. Sadness is identified with the highest accuracy of 96.69%, while disgust is recognized with the lowest accuracy of 86.3%. Disgust and anger are two facial expressions that are easily misclassified from each other. For the testing samples with anger, 8.52% of them are misclassified as disgust. Meanwhile, 6.45% of testing samples with disgust are misclassified as anger. Figure 10 illustrates 12 randomly selected testing samples in the Oulu-CASIA dataset that are misclassified by our method. Similar to the misclassified samples in CK+, most of these misclassified samples are with weak expressions, which are even quite difficult to be classified correctly by human beings.

In order to evaluate the effect of the facial expression generator, we use only 1 DiffNet to compare the test image with the neutral reference image, and the performance on Oulu-CASIA decreases from 91.3% to 80.4%.

**Figure 11:**
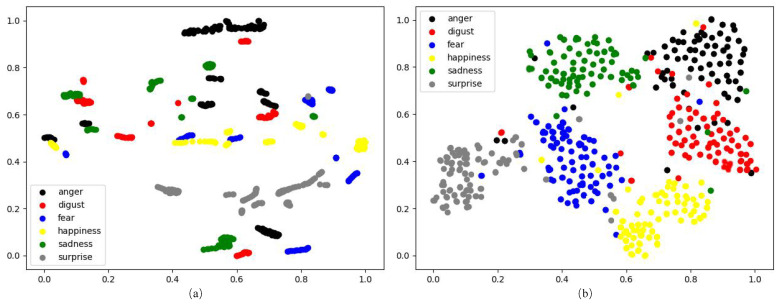
Visualization of deep features output by the VGG-face [8] (**a**) and our method (**b**) on the Oulu-CASIA dataset. Each dot represents the deep feature extracted from a testing sample.

The deep features of all the 654 testing samples in Oulu-CASIA extracted by our method and the VGG-face [8] are visualized using t-SNE [9] in Figure 11. Similar to what happened on the CK+ dataset, the deep features of all the six facial expression classes output by the VGG-face are heavily entangled, while most of the deep features output by our method are clustered according to their facial expression classes.

## 5. Conclusions

In this paper, a self-difference convolutional network (SD-CNN) is proposed for facial expression recognition. First, the SD-CNN uses a conditional generative adversarial network to generate the six typical facial expressions for the same subject in the testing image. Second, six compact and light-weighted difference-based CNNs, called DiffNets, are designed for classifying facial expressions. Each DiffNet extracts a pair of deep features from the testing image and one of the six synthesized expression images and compares the difference between the deep feature pair. In this way, any potential facial expression in the testing image has an opportunity to be compared with the synthesized “Self”. As most of the self-difference features of the images with the same facial expression will gather tightly in the feature space, the intra-class variation issue is significantly alleviated. Our SD-CNN has been extensively evaluated on two widely-used FER datasets (i.e., the CK+ and Oulu-CASIA datasets). Experimental results show that our SD-CNN achieves accuracies of 99.7% on CK+ and 91.3% on Oulu-CASIA. Without exploiting the spatial-temporal information, the SD-CNN outperforms the state-of-the-art static image-based methods and even most of the sequence-based methods on both the two datasets. Moreover, the model size of the online part of our SD-CNN is only 9.54 MB, which is much smaller than the recent deep-learning-based FER methods. In future work, we will upgrade our SD-CNN to jointly identify facial expression class and estimate facial expression intensity.

## Figures and Tables

**Table 1:** Distribution of the test images in the CK+ and Oulu-CASIA.

	Anger	Disgust	Fear	Happiness	Sadness	Surprise
CK+	13.07%	20.07%	10.68%	24.35%	10.20%	21.63%
Oulu-CASIA	17.58%	15.18%	17.68%	17.60%	15.73%	16.22%

**Table 2:** Comparisons with state-of-the-art methods on the CK+ datasets.

Methods	ACC(%)	Image/Sequence
SD-CNN (Ours)	**99.7**	Image
DeRL [12]	99.3	Image
FN2EN [18]	98.6	Image
FMPN [28]	98.0	Image
VGG-face (fine-tuned) [8]	94.9	Image
GoogLeNet (fine-tuned) [17]	95.3	Image
MicroExpNet [34]	96.9	Image
MultiAttention [24]	96.4	Image
DSAE [26]	95.8	Image
GCNet [40]	97.3	Image
DynamicMTL [41]	99.1	Image
CompactCNN (frame-based) [30]	97.4	Image
IA-gen [20]	96.6	Image
FAN [31]	**99.7**	Sequence
FAN(w/o attention) [31]	99.1	Sequence
CompactCNN [30]	98.5	Sequence
DTAGN(Joint) [29]	97.3	Sequence
CPPN [27]	98.3	Sequence
DPND [10]	94.4	Sequence
PPDN [11]	99.3	Sequence

**Table 3:** Comparisons with state-of-the-art methods on the Oulu-CASIA datasets.

Methods	ACC(%)	Image/Sequence
SD-CNN (Ours)	**91.3**	Image
FN2EN [18]	87.7	Image
DeRL [12]	88.0	Image
GoogLeNet (fine-tuned) [17]	79.2	Image
VGG-face (fine-tuned) [8]	72.5	Image
GCNet [40]	86.4	Image
DynamicMTL [41]	89.6	Image
MicroExpNet [34]	85.8	Image
MultiAttention [24]	80.2	Image
IA-gen [20]	88.92	Image
PPDN [11]	84.6	Sequence
DPND [10]	75.3	Sequence
DTAGN(Joint) [29]	81.5	Sequence
LOMO [42]	82.1	Sequence
CompactCNN [30]	**88.6**	Sequence
